# Affective temperament polygenic risk scores predict depression: investigating the role of environmental factors

**DOI:** 10.1192/j.eurpsy.2023.758

**Published:** 2023-07-19

**Authors:** D. Győrik, D. Torok, B. Erdelyi-Hamza, Z. Gal, N. Eszlari, G. Bagdy, G. Juhasz, X. Gonda

**Affiliations:** 1 Department of Pharmacodynamics; 2 Doctoral School of Mental Health Sciences; 3 Department of Psychiatry and Psychotherapy; 4 NAP3.0 Neuropsychopharmacology Research Group; 5 SE-NAP-2 Genetic Brain Imaging Migraine Research Group, Hungarian Brain Research Program, Semmelweis University, Budapest, Hungary

## Abstract

**Introduction:**

Depressive disorders are known heterogeneous both in their clinical manifestations and etiopathophysiology. Affective temperaments have a strong biological background and heritability, manifest at early age and remain stable throughout the life span, and have a pathoplastic effect in depression. Thus, they have been suggested as intermediate phenotypes for depression.

**Objectives:**

Our aim was to investigate if polygenic risk scores (PRS) calculated for the five affective temperaments predict depression and to examine their interaction effects of early and recent stressors.

**Methods:**

1820 nonrelated participants from a general population were genotyped and provided data on current depression (Brief Symptom Inventory-BSI), early (Childhood Trauma Questionnaire, CHA) and recent stressors (List of Threatening Life Events, RLE), and affective temperaments (Temperament Evaluation of Memphis, Pisa, Paris and San Diego, TEMPS-A). Our previously performed TEMPS-A GWAS analysis was used as discovery sample and the NewMood database as target sample for analysing the effects of affective temperament PRS on depression. Linear regression models were used to calculate the interaction effect of early and recent stressors.

**Results:**

PRSs derived from anxious, cyclothymic, depressive, and irritable temperaments had a significant effect on current depression, explaining 2.6-7.1% of variance. PRSs calculated from the anxious, depressive and hyperthymic temperaments significantly predicted current depression in interaction with CHA, explaining 10% of variance. In case of interaction models including both early and recent stressors, a significant effect of depressive PRS was found. Detailed results are shown in Table 1.

**Table:** 

	anxious	cyclothymic	depressive	hyperthymic	irritable	
on BSI-depression	R^2^	**.0033**	**.0071**	**.0032**	.0016	**.0026**
	p-value	**.011**	**.0002**	**.011**	.076	**.022**
in interaction with CHA	R^2^	**.1062**	.1037	**.1029**	**.1015**	.1022
	p-value	**.008**	.551	**.027**	**.038**	.531
in interaction with RLE	R^2^	.0365	.0402	.0362	.0369	.0368
	p-value	.396	.140	.483	.227	.480
in interaction with CHA and RLE	R^2^	.1387	.1384	**.1395**	.1344	.1348
	p-value	.101	.400	**.0009**	.981	.930

**Conclusions:**

Our results confirm the genetic association between affective temperaments and depressive symptoms, which highlight their role as possible clinically relevant intermediate phenotypes for depression.

**Disclosure of Interest:**

None Declared

